# Bringing memory fMRI to the clinic: Comparison of seven memory fMRI protocols in temporal lobe epilepsy

**DOI:** 10.1002/hbm.22726

**Published:** 2015-03-02

**Authors:** Karren Towgood, Gareth J. Barker, Alejandro Caceres, William R Crum, Robert D.C. Elwes, Sergi G. Costafreda, Mitul A. Mehta, Robin G. Morris, Tim J. von Oertzen, Mark P. Richardson

**Affiliations:** ^1^ Department of Basic and Clinical Neuroscience Institute of Psychiatry, Psychology and Neuroscience, King's College London London United Kingdom; ^2^ Department of Neuroimaging Institute of Psychiatry, Psychology and Neuroscience, King's College London London United Kingdom; ^3^ Center for Research in Environmental Epidemiology (CREAL) Barcelona Spain; ^4^ Centre for Epilepsy King's College Hospital NHS Foundation Trust London United Kingdom; ^5^ Department of Old Age Psychiatry Institute of Psychiatry, Psychology and Neuroscience, King's College London London United Kingdom; ^6^ Department of Psychology Institute of Psychiatry, Psychology and Neuroscience, King's College London London United Kingdom; ^7^ Department of Neurology Wagner‐Jauregg Neuroscience Centre Linz, Austria; St. George's Hospital London United Kingdom

**Keywords:** temporal lobe epilepsy, fMRI, reliability, reproducibility

## Abstract

fMRI is increasingly implemented in the clinic to assess memory function. There are multiple approaches to memory fMRI, but limited data on advantages and reliability of different methods. Here, we compared effect size, activation lateralisation, and between‐sessions reliability of seven memory fMRI protocols: Hometown Walking (block design), Scene encoding (block design and event‐related design), Picture encoding (block and event‐related), and Word encoding (block and event‐related). All protocols were performed on three occasions in 16 patients with temporal lobe epilepsy (TLE). Group T‐maps showed activity bilaterally in medial temporal lobe for all protocols. Using ANOVA, there was an interaction between hemisphere and seizure‐onset lateralisation (*P* = 0.009) and between hemisphere, protocol and seizure‐onset lateralisation (*P* = 0.002), showing that the distribution of memory‐related activity between left and right temporal lobes differed between protocols and between patients with left‐onset and right‐onset seizures. Using voxelwise intraclass Correlation Coefficient, between‐sessions reliability was best for Hometown and Scenes (block and event). The between‐sessions spatial overlap of activated voxels was also greatest for Hometown and Scenes. Lateralisation of activity between hemispheres was most reliable for Scenes (block and event) and Words (event). Using receiver operating characteristic analysis to explore the ability of each fMRI protocol to classify patients as left‐onset or right‐onset TLE, only the Words (event) protocol achieved a significantly above‐chance classification of patients at all three sessions. We conclude that Words (event) protocol shows the best combination of between‐sessions reliability of the distribution of activity between hemispheres and reliable ability to distinguish between left‐onset and right‐onset patients. *Hum Brain Mapp 36:1595–1608, 2015*. © **2015 The Authors Human Brain Mapping Published by Wiley Periodicals, Inc.**

## INTRODUCTION

New treatments reach clinical practice via a demanding process of clinical trials, in order that their is a robust evidence base to support their use. New diagnostic or prognostic investigations typically have not been subject to a similar evaluation process. Nonetheless, a clinical investigation of inadequate validity has potential for considerable harm, hence formal evaluation may be required. Examples of such formal evaluation in the neuroimaging field include evaluation of hippocampal atrophy as be a biomarker of early Alzheimer's Disease (eg., [Jack et al., 2005]), and lesion load as a surrogate marker of treatment response in multiple sclerosis (eg. [Ciumas et al., 2008]).

Surgical removal of the seizure‐onset zone is an established treatment for medial temporal lobe epilepsy (mTLE) [Wiebe et al., 2001], but carries a high risk of postoperative memory impairment of variable severity [Bell et al., 2011]. Understanding this variability and developing a method to reliably predict those at greatest risk is essential to improve presurgical planning. Measurement of hippocampal volume [Trenerry et al., 1993], preoperative memory performance [Helmstaedter and Elger, 1996; Jokeit et al., 1997] and the intracarotid amobarbital test (IAT) [Jokeit et al., 1997; Lee et al., 2003; Loring et al., 1995] are imperfect predictors of postoperative memory decline [Dupont et al., 2010]. In addition, IAT may pose risks of embolism and intra‐arterial injury [Bendszus et al., 2004; Haag et al., 2008]. Neuroimaging in presurgical evaluation of treatment‐resistant focal epilepsies has been widely studied, to increase the sensitivity and specificity of anatomical localisation of the seizure onset zone, and to quantify the risk of adverse postoperative cognitive outcome, especially language and memory outcome in patients with mTLE undergoing surgery. Disappointingly, meta‐analysis has failed to find adequate evidence to support the role of any neuroimaging technique in presurgical decision‐making [Burch et al., 2012; Whiting et al., 2006].

In addition to structural techniques, there is are specific ways in which functional magnetic resonance imaging (fMRI) may contribute to epilepsy surgical planning: first, fMRI might localise or lateralise important brain functions and predict the postsurgical outcome for these functions; second, fMRI might indicate the presence of localized or lateralized dysfunction which might indicate a functional deficit zone or the seizure onset zone. Either of these contributions could have significant implications for surgical decision‐making, and hence should be supported by an adequate evidence‐base. Contributing to the development of this evidence‐base is the primary aim of the current study. A number of studies have demonstrated predictive value of memory fMRI for lateralization of the seizure onset zone and for postoperative memory outcome in mTLE. At the time of designing the current study, three groups had independently demonstrated predictive value for postoperative memory outcome, using substantially different protocols, in relatively small “diagnostic accuracy” studies. One approach presented a series of items to be encoded into memory during scanning, initially using only verbal items [Richardson et al., 2006, 2004b] and later combining verbal and nonverbal stimuli [Bonelli et al., 2010; Powell et al., 2005, 2007, 2008]. A second approach presented complex visual scenes during scanning [Mechanic‐Hamilton et al., 2009; Rabin et al., 2004], and a third approach used in‐scanner subjective visualisation of familiar local journeys [Janszky et al., 2005]. We based the current study on these protocols, but recognise that recent studies using different methods have also shown prediction of postoperative memory outcome for example, [Binder et al., 2008, 2010; Dupont et al., 2010; Frings et al., 2008].

The emergence of multiple methods poses challenges and uncertainties. In the context of validation of hippocampal atrophy as a marker of early Alzheimer's Disease, a variety of techniques to measure hippocampal volume have demonstrated substantially similar predictive value; hence it is proposed [EMA, 2011] that individual groups may validly use their favoured method. However, memory fMRI does not show similar predictive value for postsurgical memory decline in mTLE across the multiple methods cited above. For example, even within one class of methods, the anatomical location of the brain region with the strongest predictive value for verbal memory outcome has been inconsistent, varying by up to 24 mm (compare [Richardson et al., 2004a] with [Powell et al., 2008]) and being very variable in predictive value, with correlation coefficient between fMRI measures and postsurgical memory decline varying between 0.23 [Bonelli et al., 2010] and 0.92 [Powell et al., 2008]. Furthermore, the detection of fMRI signal in the relevant mesial temporal region can be difficult due to artefacts caused by susceptibility effects, with the consequence that activations may be located on the margins or within low‐signal areas which are likely to be noisy and unreliable. It is plausible that an important source of disagreement between studies is measurement noise, hence in this context a careful examination of the measurement stability of memory fMRI over multiple sessions is necessary.

Missing from current evidence is a rigorous examination of the comparative effect size, activation lateralisation and reliability of a range of memory fMRI tasks in mTLE patients. Without this, the subsequent clinical application of memory fMRI in mTLE may be flawed. Here, we compare head‐to‐head seven fMRI protocols in a group of patients with mTLE. We see this as a much‐needed step in bringing memory fMRI into clinical practice. We aim to answer five questions:

1. Can mTLE patients perform these tasks? We examine fMRI task‐related behaviour, to establish whether mTLE patients can reliably perform the tasks.
2. Is one protocol more strongly activating, or more strongly lateralising, than another? Using ANOVA of the mean effect sizes over a mTL ROI for each subject, protocol and session, we examine whether variability in the data is accounted for by protocol, session, hemispheric lateralisation of activation, or seizure‐onset lateralisation (or interactions between these factors).
3. Using ICC applied to voxelwise effect size data, are these protocols reliable across sessions in terms of magnitude of activation?
4. Using between‐sessions overlap of voxelwise effect size, are these protocols reliable in terms of the anatomical localisation of activation?
5. On the basis of asymmetry of mean effect size in right and left mTL ROIs, are these protocols reliable in terms of lateralisation of activation, and do these protocols permit classification of mTLE patients into those with seizure onset on the right and onset on the left?

We intend that our findings will allow the comparative activation effect size within the mTL ROI, effectiveness of lateralisation and reliability of these protocols to be clarified. These findings may then inform the design of much‐needed prospective clinical trials to predict cognitive outcomes of surgery in mTLE, as recently called for by the UK National Institute of Health Research, and act as benchmarks for the development of superior fMRI methods in the future.

## MATERIALS AND METHODS

### Participants

We studied 16 right handed participants with mTLE recruited from King's College Hospital and St. George's Hospital, London, UK. Inclusion criteria were: clinical diagnosis of mTLE, aged ≥18, full scale IQ ≥70, no known history of significant head injury, other neurological disorders or previous psychiatric history, literate in English, normal vision (corrected as needed), able to give informed consent and no contraindications to MRI. Research Ethics Committee approval was obtained prior to commencing the study, and all participants gave written informed consent.

### Study Design

Each of the 16 participants completed three sessions (referred to as T1, T2, and T3), with each session separated by at least 2 weeks. Each session involved three fMRI studies. To allow familiarization with equipment and tasks, and to minimize practice and learning effects, before the first session each participant completed a practice session outside the scanner.

### Hometown Walking Study

The Hometown Walking study shows brain regions active while imagining a familiar route compared with a low level control task, as a block design. We replicated a previously described study [Janszky et al., 2005]. Prior to the first session, participants were instructed to prepare an imagined walk starting and ending at familiar places, passing participant‐defined waypoints en route. Participants were instructed to divide the route into 10 segments, with each waypoint as the starting point for the next segment. Each participant used the same imagined walk for each of the three sessions. The fMRI paradigm consisted of 10 task blocks and 10 control blocks. Each block was 30 sec in duration, with the total task lasting 10 min. During scanning, participants were cued on the screen to imagine each segment of the walk during activation blocks, and to count up silently in odd numbers from 21 during the control blocks.

### Visual Scenes Study

The Visual Scenes study shows brain regions active while viewing complex visual scenes versus a visual control condition of “scrambled” scenes, presented in blocks, replicating a previously described study [Rabin et al., 2004]. This task can be analysed as a block design, or as an event‐related design based on a postscan recognition test. The fMRI paradigm consisted of 7 task and 7 control blocks, with each pair of blocks preceded by a 20s cross hair fixation. Each task block consisted of nine complex visual scenes presented on the screen for 3,600 ms, followed by 400 ms of blank screen. The baseline blocks consisted of a single “scrambled” image, comprising one of the scene images degraded using a random‐retiling algorithm. The total task lasted 9 min. During scanning, participants were instructed to indicate with a button‐press if they liked or disliked each scene or scrambled image. They were also asked to memorise the scenes for a recognition test. After scanning, memory was evaluated with a self‐paced “seen”/“unseen” recognition test. The 63 complex scenes presented during the encoding task were presented again intermixed with 35 new complex visual scenes as foils. The task was presented on a laptop computer and the participants used the keyboard to indicate whether each item was “seen” or “unseen.”

### Words and Pictures Study

The Words & Pictures study shows brain regions active during the viewing of words or pictures, presented in blocks, compared with a low level control condition. The data can be analysed as a block design, or as an event‐related design based on a postscan recognition test. This study is based on a modification of the study described previously [Powell et al., 2005]. We omitted the face encoding condition because of poor recognition memory for these stimuli in a previous study [Powell et al., 2005], and greatly divergent anatomical localisation of activity between studies (compare [Powell et al., 2008] with [Bonelli et al., 2010]). We note also recent evidence for poor reliability of BOLD activation for face stimuli [Plichta et al., 2012].

The pictures were presented as colour images. The words were concrete nouns with a length between 3 and 8 characters and a mixture of neutral and emotional words. The fMRI paradigm consisted of alternating blocks of words and pictures, with each of the 14 blocks preceded by a 20 sec cross hair fixation. Each word block consisted of 10 words (8 neutral and 2 emotional) presented on screen for 3,500 ms, followed by 2,000 ms of blank screen. Each of the picture blocks consisted of 10 pictures presented on screen for 3,500 ms, followed by 2,000 ms of blank screen. Participants were instructed to indicate with a button‐press if they liked or disliked each word or picture. They were also asked to memorise the words and pictures for a recognition test. The total task lasted just over 15 min. The material order was counterbalanced across subjects. Three sets of stimuli were created, so that different stimuli were presented at each session, matched according to image size for pictures and written word frequency [Coltheart, 1981; Kucera and Francis, 1967].

The order in which in‐scanner paradigms were undertaken was pseudorandomised across subjects to avoid order effects, but between‐sessions within‐subjects the order was held constant. After the scanning session was completed, memory was evaluated with a self‐paced “seen”/“unseen” recognition test. The 70 words and 70 pictures presented during scanning were presented again intermixed with 42 new words and 42 new pictures as foils.

### Image Acquisition

Conventional gradient‐echo echo‐planar imaging (EPI) fMRI was used, with an acquisition designed to maximise visualization of the temporal lobes (SIGNA HDx 3.0T MR scanner, General Electric, Milwaukee WI), using the body coil for transmission and an 8 channel head coil for reception. Twenty‐nine slices of EPI data were acquired (sequential slices superior to inferior, repetition time (TR) 2,500 ms, echo time (TE) 30 ms, flip angle 80°, slice thickness 1.9 mm, gap 0.1 mm, matrix 64 × 64, field of view (FOV) 180 mm). Images were acquired parallel to the long axis of the hippocampus, and positioned to maximise the coverage of the temporal lobes given the restricted (6 cm) coverage in the inferior/superior direction and small field of view. An example EPI volume is shown in Supporting Information Figure 1. To allow registration of fMRI data to standard space, a whole‐brain EPI data set comprising 80 slices in the same orientation but with increased in‐plane and through‐plane coverage was also acquired (TR 7,000 ms, TE 30 ms, flip angle 90°, slice thickness 1.9 mm, gap 0.1 mm, matrix 64 × 64, FOV 220 mm).

### Behaviour Analysis

Data were collected for response type (“like”/“dislike”) and for number of missed responses, for each subject, fMRI task and session. Reliability of behavioural scores was estimated using two‐way mixed model ICC, Model 3 [Shrout and Fleiss, 1979]. Different guidelines exist for the interpretation of the ICC. Here we take an ICC value of less than 0.40 to be poor, 0.40–0.59 as fair, 0.60–0.74 as good and values >0.75 as excellent [Fleiss et al., 2003]. These terms should be interpreted with caution as they do not take into account the confidence intervals of the ICC measure.

Recognition accuracy was calculated as hit rate minus false alarm rate, for each participant, session and task, and entered into a two‐way repeated measures ANOVA. Additionally, reliability of recognition accuracy was estimated using two‐way mixed model ICC, Model 3 [Shrout and Fleiss, 1979]. All behavioural data were analysed with IBM SPSS Statistics 19.

### Image Processing

The functional data were analysed using SPM8 software (http://www.fil.ion.ucl.ac.uk/spm). For each participant, EPI time series data for each study and session were realigned using the mean image for that study and session, slice time corrected, coregistered with the whole brain EPI image from session T1, volumetrically normalized to the EPI template provided with SPM, and smoothed with an isotropic 6 mm full‐width‐half‐maximum Gaussian kernel. The robust weighted least squares method [Diedrichsen and Shadmehr, 2005] method was used to detect and adjust for noise and movement artefacts. We chose not to exclude participants due to excessive movement, to reflect the aim to establish clinical reliability in real‐world application. For block design analysis of the Hometown, Scenes, and Words & Pictures studies, regressors of interest were formed for each protocol by creating a single boxcar function convolved with the canonical hemodynamic response function. Contrast images for each protocol were created to compare the study activation to the control condition. An additional event related design analysis was also conducted for the Scenes and Words & Pictures studies. We examined here contrast images of remembered items versus baseline. In summary, we examined seven protocols:

1. Hometown, block design.
2. Scenes, block design.
3. Scenes, event‐related design.
4. Pictures, block design.
5. Pictures, event‐related design.
6. Words, block design.
7. Words, event‐related design.

### Initial Characterisation and Description of the Data

For each protocol we performed a conventional second‐level one‐sample *T*‐test in SPM8 using the contrast images described above, separately for each session, to confirm medial temporal lobe (mTL) activity was present. Next, we took the beta parameter images for each subject, session and protocol. From these images we extracted voxelwise effect‐size (beta parameter) data for a large mTL . An anatomical mTL (ROI) (Supporting Information Figure 2) was created by combining the right and left hippocampus, amygdala and parahippocampal gyri from the Harvard‐Oxford Structural Atlas [Kennedy et al., 1998; Makris et al., 1999] provided in the FSL software (http://www.fmrib.ox.ac.uk/fsl/). The voxelwise beta parameters for each subject (thresholded at *P* < 0.001), session and protocol within each half of the mTL ROI (right hemisphere, left hemisphere) were averaged. We tested the hypothesis that there would be effects of session, hemisphere, protocol and seizure‐onset lateralisation using a conventional repeated‐measures ANOVA. Within‐subjects factors were session (3 levels), hemisphere (2 levels) and protocol (7 levels), and between‐subjects factor was seizure‐onset lateralisation (2 levels).

### Establishing Reliability of Magnitude of Activation

Intraclass correlation coefficient (ICC) is defined as the ratio of the between‐subject variance to the total variance [Shrout and Fleiss, 1979] and is higher when between‐subject variance is high and within subject‐variance is low [Clement and Belleville, 2009]. A number of authors have found ICCs to be a useful measure of reliability in fMRI studies [Aron et al., 2006; Bennett and Miller, 2013; Clement and Belleville, 2009; Fernandez et al., 2003; Manoach et al., 2001; Raemaekers et al., 2007]. We used the voxel‐wise calculation of ICC [Caceres et al., 2009].

A two‐way mixed ICC model (Model 3 [Shrout and Fleiss, 1979]) was used to index reliability of activation over sessions within the mTL ROI. This model includes subject as a random effect and session as a fixed effect. Two different reliability measures were calculated: The first, ICC_MED_ [Caceres et al., 2009] is based on the median of the voxelwise ICC distribution within the ROI. The second measure, ICC_V_, is an intravoxel measurement to determine the reliability across sessions of the distribution of ICC values within the ROI and is calculated per subject. These measures of reliability can be thought of as measuring the consistency of the rank‐ordering of the participants (ICC_MED_), or voxels within an ROI (ICC_V_). We applied ICC_MED_ and ICC_V_ to the voxelwise data from the mTL ROI for each protocol across sessions.

### Establishing Reliability of Localisation of Activation

The spatial consistency of activated voxels across visits was assessed using two measures of generalized spatial overlap [Crum et al., 2006] which extend standard Dice overlaps to summary measures over all subjects, and can include overlaps of gray‐level as well as binary data. The spatial consistency of significant activation was assessed from binary maps of voxels thresholded for significance at *P* < 0.001. In addition, the spatial consistency of the pattern of activation was assessed from the raw t‐images individually normalized to the peak *t*‐value within a [Voyvodic et al., 2009]. We applied these two measures of spatial overlap to the activated voxels in the ROI for each protocol across sessions.

### Establishing Reliability of Laterality of Dysfunction

The determination of hemispheric lateralisation of memory is potentially an important clinical application for fMRI in epilepsy [Bonelli et al., 2010; Richardson et al., 2004a]. Hemispheric dominance in fMRI is most commonly calculated through a laterality index or asymmetry index. For the present study we calculated asymmetry index values for all participants for whom seizure onset could be lateralized from clinical data, using the mTL ROI as described above, divided into symmetrical halves by the midsagittal plane. Asymmetry indexes were calculated using the mean effect size statistic (*β* parameter) for the activated voxels, thresholded at *P* < 0.001, for each of the seven protocols described above, using the following formula:

Asymmetry index = β(ipsilateral mTL ROI) – β(contralateral mTL ROI)

(Note that the beta parameter is a normalized value so does not need to be further normalized by dividing the difference by the sum or mean.) This method has been used previously to show prediction of postoperative verbal memory decline in mTLE patients undergoing surgery using preoperative asymmetry of verbal encoding activity in the mTL [Bonelli et al., 2010; Richardson et al., 2004a].

In addition, we examined the receiver operating characteristic (ROC) curves to examine the ability of asymmetry index to classify participants by seizure onset laterality, for each of the seven protocols. ROCs plot the true positive rate (sensitivity) as a function of its false positive rate (specificity) across a range of threshold values of the test metric, and can be used for evaluating the accuracy of classifying patient groups using a diagnostic test [Altman and Bland, 1994]. As a final exploratory analysis, we examined whether repeating the same protocol over multiple sessions improves classification accuracy. We examined the effect of averaging within‐subjects the asymmetry indexes from the first two sessions (T1 and T2) and also the effect of averaging across all three sessions (T1, T2, T3). ROC analysis was performed on the averaged asymmetry indexes.

## RESULTS

### Participants

Sixteen participants were enrolled (Supporting Information Table I). Patients 1 and 2 did not complete the Words and Pictures studies at T1, and patient 1 did not complete the Words and Pictures studies at T2. Patient 14 showed a significant task by motion effect during one session for the Hometown Walking Task, hence these data could not be analysed. We therefore report results for 13 patients where we have complete data. We also report findings for the full dataset of 16 patients in Supporting Informations. For patient 16, it was not possible to lateralise the side of seizure onset, therefore this subject is excluded from examination of asymmetry index.

### Behaviour

All patients were able to perform the tasks. All participants responded with both “Like” and “Dislike” responses suggesting that all were actively attending to the tasks (Supporting Information Table II). Reliability of percentage “Like” response across sessions was fair for Scenes ICC_(3,1)_ 0.572 (*P* < 0.001), good for Pictures 0.747 (*P* < 0.001), and fair for Words 0.429 (*P* < 0.008). Reliability of missed response rate across sessions was excellent for Scenes 0.895 (*P* < 0.001), good for Pictures 0.656 (*P* < 0.001), but poor for Words 0.327 (*P* < 0.029).

**Figure 1:**
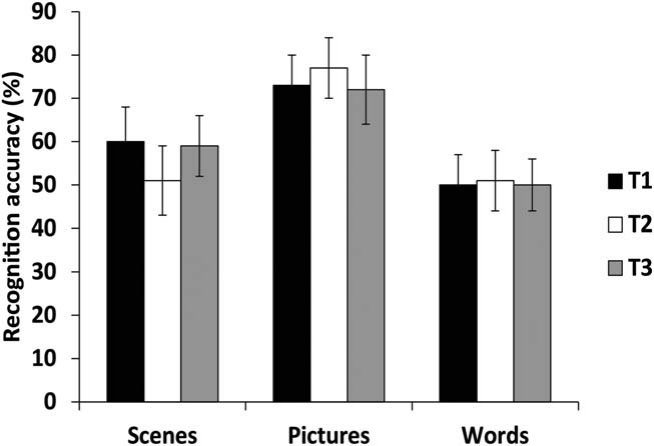
Mean recognition accuracy for participants across session and fMRI protocol. Recognition accuracy is calculated as hit rate minus false alarm rate. Error bars represent 1 SEM.

There were no significant differences in recognition accuracy for Scenes, Pictures or Words across sessions (*F* = 0.102, *P* = 0.903, Fig. 1). There were however differences in recognition accuracy between tasks (*F* = 12.782, *P* < 0.001). Post hoc *t*‐tests suggest recognition accuracy was better for Pictures than Scenes (*t* = −6.046, *P* < 0.001) and better for Pictures than Words (*t* = 7.461, *P* < 0.001) but was not different for Scenes compared with Words (*t* = 1.764, *P* = 0.102). For each of the tasks, the reliability of recognition accuracy across the three sessions was excellent for Pictures ICC_(3,1)_ 0.776 (*P* < 0.001), excellent for Scenes 0.836 (*P* < 0.001) and fair for Words 0.527 (*P* < 0.001).

### Initial Characterisation and Description of the Data

Thresholded group t‐maps for each of the T1 protocols are shown in Supporting Information Figure 3. Activity is evident bilaterally in mTL at the group level for all protocols. There was no main effect of session and no interactions involving session, suggesting activation did not differ across T1, T2, and T3 for any protocol or patient subset (Table 1). There was no main effect of hemisphere; given that our patient group was roughly balanced between right‐onset and left‐onset, and that we hypothesised that lateralisation of activation would favour the contralateral side, finding no effect of hemisphere across the whole group is not unexpected. However, there was a strong interaction between hemisphere and seizure‐onset lateralisation, suggesting that lateralisation of activity is different between left‐onset and right‐onset cases. There was a strong effect of protocol, suggesting that the strength of mTL activation is different between protocols. Furthermore, there was an interaction between hemisphere and protocol, suggesting that protocols differed in the extent to which left and right hemispheres were activated. Finally and crucially, there was an interaction between hemisphere, protocol and seizure‐onset lateralisation, revealing that the difference in activity between left and right hemispheres in left‐onset and right‐onset groups differed between protocols. This finding suggests that these protocols may not be equal in their ability to differentiate between left‐onset and right‐onset patients, which is further explored below.

**Table 1:** Effects revealed by repeated‐measures ANOVA

Source	*F*	Sig.
Session	0.272	0.765
Session × seizure‐onset lateralisation	0.036	0.965
Hemisphere	1.745	0.216
Hemisphere × seizure‐onset lateralisation	10.309	0.009
Protocol	19.711	0.000
Protocol × seizure‐onset lateralisation	0.781	0.588
Session × hemisphere	0.693	0.512
Session × hemisphere × seizure‐onset lateralisation	1.711	0.206
Session × protocol	0.844	0.606
Session × protocol × seizure‐onset lateralisation	1.290	0.233
Hemisphere × protocol	2.425	0.036
Hemisphere × protocol × seizure‐onset lateralisation	3.930	0.002
Session × hemisphere × protocol	0.534	0.889
Session × hemisphere × protocol × seizure‐onset lateralisation	1.088	0.377

Averaged beta‐parameter values for right and left mTL ROIs were examined, for each subject, session and protocol.

Note that although above we explored averaged beta parameters for left and right mTL ROIs, the effects demonstrated are identical in an analogous ANOVA using asymmetry indexes for each subject, session and protocol, and with session, protocol and seizure‐onset lateralisation as factors. We regard this as a further justification for our emphasis on the use of asymmetry index to classify patients (further explored below).

### Establishing Reliability of Magnitude of Activation

**Figure 2:**
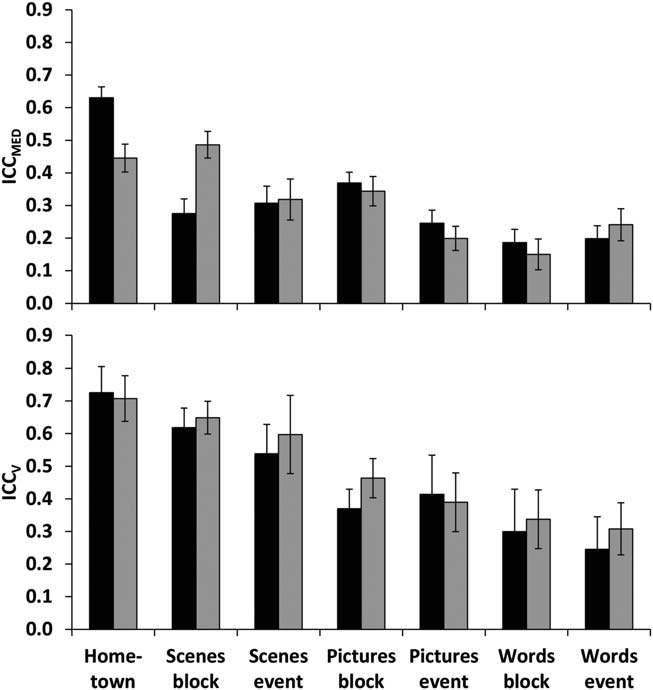
Reliability of medial temporal lobe ROI BOLD signal activation for each fMRI protocol. Black bars show comparison of T1 with T2, and gray bars show comparison of T2 with T3 (*n* = 13). Here we take an ICC value of less than 0.40 to be poor, 0.40–0.59 as fair, 0.60–0.74 as good and values exceeding 0.75 as excellent. Error bars show SEM. The upper panel shows ICCmed and the lower panel shows ICCv. ICC = intraclass correlation coefficient, ICCmed and ICCv are defined in the Methods section.

A voxelwise map of the anatomical distribution of the group ICC measure for the T2 to T3 comparison for each of the protocols is shown in Supporting Information Figure 4. The ICC values for each protocol are illustrated in Figure 2 (*n* = 13). The ICC data for each protocol for the full data set (*n* = 16) are reported in Supporting Information, and were extremely similar (Supporting Information Fig. 5). Broadly, the Hometown protocol was most reliable (good reliability using ICC_V_ and fair reliability with ICC_MED_), then in order Scenes (fair to good reliability using ICC_V_ and poor to fair reliability with ICC_MED_), Pictures (mostly poor reliability) and Words (poor reliability); the differences between block and event‐related analyses were not striking.

We explored within‐subjects between‐sessions reliability in more detail. We calculated the mean beta parameter (thresholded at *P* < 0.001) within each half of the mTL ROI, dividing into ipsilateral and contralateral mTL ROIs for all subjects, protocols and sessions. These data are illustrated in Supporting Information Figures 6 and 7. This reveals two key findings. First, effect sizes are typically considerably greater for event‐related than block analyses, for the protocols evaluated here. Second, the within‐subjects between‐sessions variability of mean ROI beta parameter is considerably greater for those tasks with larger effect sizes. We conclude from this, for the protocols studied here, that tasks producing small effects are highly reliable but tasks producing large effects may be less reliable.

### Establishing Reliability of Localisation of Activation

**Figure 3:**
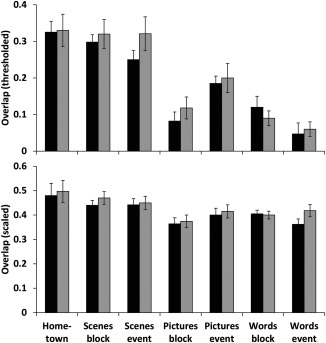
Overlap values of activated voxels in medial temporal lobe ROI for each fMRI protocol (*n* = 13). Black bars show overlap values comparing T1 with T2; gray bars show overlap values comparing T2 with T3. The upper panel shows overlap of binary maps of voxels thresholded for significance at *P* < 0.001, within the MTL ROI, by task; the lower panel shows overlap values generated from raw t‐images individually normalized to peak t values within the medial temporal lobe ROI.

Figure 3 displays the overlap values for the binary maps of voxels within the bilateral mTL ROI thresholded for significance at *P* < 0.001, and the overlap values for the t‐images individually normalized to the peak *t*‐value within the bilateral mTL ROI. The binarised and normalized overlap values for the full data set (*n* = 16) are reported in the Supporting Information and were extremely similar (Supporting Information Fig. 8). Examining overlap of binarised voxels, the Hometown protocol and the Scenes protocols (block and event) showed the greatest between‐sessions overlap, whereas Pictures (block) and Words (block and event) tasks showed substantially less overlap. However, when the mTL ROI voxels were normalized to peak *t*‐scores, the extent of overlap was considerably improved for all protocols and was much more similar between protocols.

### Establishing Reliability of Laterality of Dysfunction

**Figure 4:**
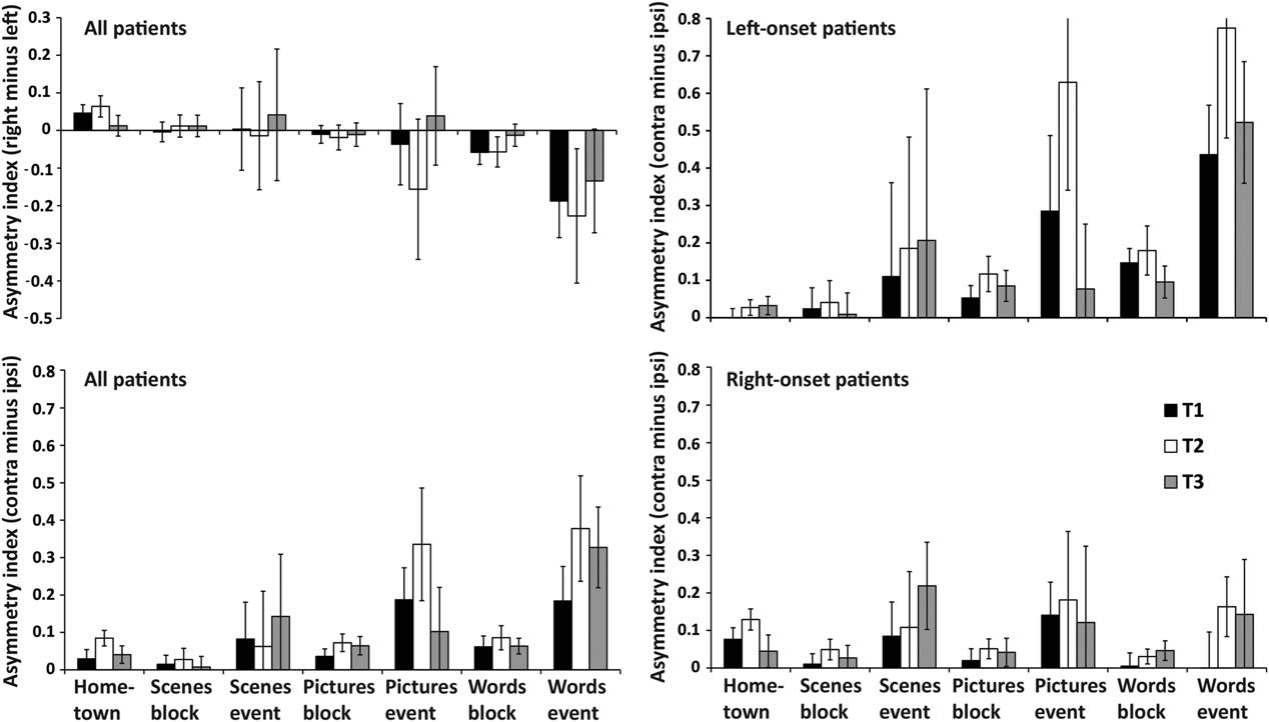
Average laterality index (*n* = 13) of mean voxel activity in each medial temporal ROI (average of voxels thresholded at *P* < 0.001) for each fMRI protocol and session. Error bars show SEM. Upper left panel shows laterality index as right‐minus‐left ROI for all patients, which may provide an indication of material‐specific lateralisation of the fMRI tasks; lower left panel shows laterality index as contralateral‐minus‐ipsilateral ROI for all patients; upper right panel shows laterality index as contralateral‐minus‐ipsilateral ROI for left‐onset patients (note this is the same as right‐minus‐left ROI); lower right panel shows laterality index as contralateral‐minus‐ipsilateral ROI for right‐onset patients (note this is the same as left‐minus‐right ROI). Black bars are for T1, white bars T2 and gray bars T3.

Table 2 shows the ICC of asymmetry index (contralateral ROI minus ipsilateral ROI) for T1 versus T2, and T2 versus T3. Scenes (event), Scenes (block) and Words (event) show the most consistent reliability of asymmetry index between sessions. Supporting Information Table III shows the same data for the full group *n* = 16, and is extremely similar. Figure 4 displays the laterality Indexes for each of the tasks and sessions; Supporting Information Figure 9 shows the same data for the whole group *n* = 16 and is extremely similar. Across the group of patients, activity for the Hometown protocol tends to be lateralized to the right mTL, and activity for the Words protocols (block and event) tends to be lateralized to the left, whereas the other protocols show no predominant lateralisation to left or right. This figure also shows that all the protocols tend to produce more activation in the contralateral than ipsilateral mTL ROI, and that this asymmetry is strongest for the Pictures protocols and Words protocols, and also stronger for event‐related analyses than block analyses.

**Table 2:** Reliability of medial temporal lobe ROI BOLD signal laterality index for each fMRI protocol

fMRI protocol	T1 compared with T2		T2 compared with T3	
	ICC_(3,1)_	Significance	ICC_(3,1)_	Significance
Hometown	0.541	0.028	0.384	0.098
Scenes‐block	0.577	0.020	0.778	0.001
Scenes‐event	0.676	0.006	0.802	0.000
Pictures‐block	0.456	0.059	0.790	0.001
Pictures‐event	0.373	0.105	0.131	0.335
Words‐block	0.575	0.020	0.352	0.119
Words‐event	0.555	0.024	0.701	0.004

Here, we take an ICC value of less than 0.40 to be poor, 0.40–0.59 as fair, 0.60–0.74 as good and values exceeding 0.75 as excellent. T1 is compared with T2, and T2 with T3 (*n* = 12). ICC = intraclass correlation coefficient, ICC_(3,1)_ is defined in the Methods section. Significance threshold set according to false discovery rate.

Therefore, and importantly, the Scenes protocols produce reliable activation, but in this patient group it is symmetrical and nonlateralising; only the Words (event) protocol shows evidence of asymmetric lateralising activation over all three time points. We illustrate these data in more detail in Supporting Information Figure 10, which shows asymmetry index for each subject, protocol and session. These data allow us to identify whether any subject showed different lateralisation between different sessions for the same protocol; these data reveal that every subject showed different lateralisation between sessions for at least one protocol. However, it may be more reasonable to consider whether “strong” lateralisation varies between sessions for the same protocol. Arbitrarily, we defined “strong” lateralisation as >1 SD from zero (either positive or negative), SD being estimated for each protocol, pooled across sessions. All but one of the 16 subjects showed strong lateralisation of activity in at least one protocol in at least one session (therefore one subject showed no strong lateralisation for any protocol in any session, patient 7). Four subjects showed a change from strong lateralisation in one direction to strong lateralisation in the opposite direction between sessions for the same protocol [patient 2 for Picture (block); patient 4 for Picture (block) and Words (event); patient 10 for Hometown; patient 14 for Words (block)].

The ability of each task to reliably identify the side of seizure onset was further explored using ROC analysis. Table 3 shows the ROC results for each protocol by session. Supporting Information Table 4 shows the same data for the full group *n* = 16, and is extremely similar. Only the Words (event) protocol was able to achieve a significantly above‐chance classification of patients at all three sessions, both for the *n* = 13 group that followed the full protocol, and for the *n* = 16 full group; area under the curve (AUC) was approximately 0.9 or higher at all sessions. Averaging data over multiple sessions led to a modest improvement in classification performance for all protocols except Scenes (block and event).

**Table 3:** Classification of patients into right‐onset and left‐onset using receiver operating characteristic

fMRI protocol	T1		T2		T3		Average T1, T2		Average T1, T2, T3	
	AUC	Sig.	AUC	Sig.	AUC	Sig.	AUC	Sig.	AUC	Sig.
Hometown	0.771	0.123	1.00	0.004	0.771	0.123	0.943	0.012	0.943	0.012
Scenes‐block	0.629	0.465	0.771	0.123	0.543	0.808	0.714	0.223	0.686	0.291
Scenes‐event	0.686	0.291	0.686	0.291	0.571	0.685	0.686	0.291	0.686	0.291
Pictures‐block	0.800	0.088	0.943	0.012	0.829	0.062	1.000	0.004	1.000	0.004
Pictures‐event	0.829	0.062	0.886	0.028	0.686	0.291	0.914	0.019	0.886	0.028
Words‐block	0.914	0.019	0.971	0.007	0.886	0.028	0.943	0.012	1.000	0.004
Words‐event	0.886	0.028	0.971	0.007	0.914	0.019	0.943	0.012	1.000	0.004

ROC was applied to the laterality index for each subject, for each fMRI protocol and session (*n* = 12). To examine whether repeating a protocol on more than one session contributes to accuracy of lateralisation, asymmetry values were averaged across T1 and T2 for each subject and protocol, and averaged across T1, T2, and T3 for each subject and protocol. AUC = area under curve.

## DISCUSSION

In the context of applying memory‐related fMRI evaluations to patients with mTLE, a key question is how memory‐related function is distributed between the right and left mTLs. Prior evidence suggests that more memory‐related activity in the mTL which is subsequently resected is associated with increased risk of postoperative memory decline [Janszky et al., 2005; Rabin et al., 2004; Richardson et al., 2004a]. Although we include in our analysis here a voxelwise detailed appraisal of activation reliability in the mTL, it is not yet plausible that epilepsy surgery could be guided by the detailed anatomy of memory‐related fMRI effects—in contrast to the findings of sensorimotor fMRI, which may provide an explicit anatomical guide to the surgeon. Hence our focus is substantially on a comparison between these seven protocols of the overall magnitude of activation, lateralisation of activation, and classification of patients into left‐onset and right‐onset groups. We do not present here data regarding prediction of postoperative outcome, and anticipate future studies will address this question. We selected fMRI protocols which had already shown some association with postsurgical outcome in previous studies. Our intention was not to replicate these studies, but instead to better characterise these tasks for future replications on a larger scale by understanding the most reliable imaging metrics.

We undertook a comprehensive examination of seven memory‐related fMRI protocols from many perspectives, and used a relevant patient group rather than normal subjects, making a number of findings. We found, on the basis of available behavioural data and plausible activation maps, that any of these seven protocols is achievable for patients with mTLE. Most task‐related behaviours were reliable across sessions for these protocols in mTLE patients. Our ICC approach to voxel‐level reliability of magnitude of activation in a large bilateral mTL ROI showed variable reliability between different protocols; in all cases reliability was somewhat modest. It was striking that protocols with largest activation effect sizes tended to have least reliability; large activation effect size is a necessity for a single‐subject fMRI study, hence this is a challenging finding for the clinician aiming to implement memory‐related fMRI in this context. Spatial overlap of anatomical localisation of activation showed very similar modest overlap and very similar relative differences between protocols as with our ICC analysis. We noted a marked difference between protocols in asymmetry of activation between right and left mTLs. The Hometown protocol is modestly right‐lateralising; Words (block and event) is more strongly left‐lateralising; other tasks do not preferentially activate right or left across the group. All protocols preferentially activate the contralateral mTL across the group of patients, though this is variable between protocols—Words and Pictures are most strikingly asymmetric, and event related analyses were more so than block designs. Asymmetry of activation is consistent between sessions, most convincingly for Scenes (block and event), Pictures (block) and Words (event) but note that Scenes (block and event) and Pictures (block) do not have either a strong left‐right or strong contralateral‐ipsilateral lateralising tendency. Using a binary classifier, only the Words (event) protocol was able to achieve a significantly above‐chance classification of patients at all three sessions. Averaging data over multiple sessions led to a modest improvement in classification accuracy. Therefore we propose that a simple asymmetry index assessed using the Word (event) protocol, possibly repeated on more than one occasion, shows most promise as a clinical tool to assess lateralisation of memory function in mTLE.

### Establishing Reliability of Magnitude of Activation

The study revealed that the nature of the memory task had an influence on reliability. The Hometown protocol showed mostly good reliability by both measures of ICC; Scenes (block) showed poor to fair reliability using ICC_MED_ and good reliability using ICC_V_; Scenes (event) showed poor reliability with ICC_MED_ and fair with ICC_V_; Words and Pictures protocols generally produced poor reliability. This is consistent with prior work [Harrington et al., 2006] showing a difference in reliability between three different memory tasks (word‐pair, pattern, and scene encoding) when using laterality indices as the outcome measure. This study also found that a scene encoding task produced greater between and within subject reliability than a word‐pair or pattern encoding task.

The block design models generally produced better reliability than the event design models. In a prior study investigating the impact of fMRI experimental design [Narayan et al., 2005], a block design model was more sensitive to detecting MTL activity, per unit of scanning time, which may be important when considering applications in clinical populations. However, event related designs may still be of clinical use if they can be shown to detect relevant effects not found in block designs. For example, in a previous investigation of a similar Words & Pictures study, an event related analysis was more sensitive to anterior hippocampal activation than a block design [Powell et al., 2005].

As has generally been found in a number of other studies [Bonelli et al., 2010; Caceres et al., 2009; Wei et al., 2004], the variance between participants across sessions was greater than the variance within individual participants across sessions. Greater between subject variability is most likely due to variability in the anatomy and function of individuals' brains [Wei et al., 2004], and is not surprising in patient groups where there may be variability in the anatomical extent and severity of pathology.

### Establishing Reliability of Localisation of Activation

Measures of between‐sessions spatial overlap of activated voxels produced similar results to the ICC reliability measures in terms of relative ranking of the memory protocols, with the Hometown and Scenes protocols generally out‐performing the Words and Pictures protocols. We found low to moderate overlap values, similar to those reported for memory tasks within the MTL in other studies [Fernandez et al., 2003; Harrington et al., 2006; Putcha et al., 2011]. It has been argued previously that spatial consistency measures may not be reliable, particularly within the hippocampus where marked individual variability in morphology is noted [Putcha et al., 2011]. Further, the low to moderate overlap values do not appear to meet the benchmark standard we would expect for detailed localisation of the anatomical regions activated by a memory task which then can be used to, for example, guide the boundaries of a surgical resection.

### Establishing Reliability of Laterality of Dysfunction

We do not propose that memory fMRI would be a useful tool in the clinic for establishing the side of seizure onset in mTLE; there are better ways to achieve this. However, given that previous studies using memory fMRI to predict postsurgical memory outcome have found that the relative distribution of activity between left and right (i.e., lateralisation) is a useful measure to predict outcome, we evaluated reliability of lateralisation here. Furthermore, an interesting question might be whether the presence of unilateral mTL pathology impacts on material‐specific lateralisation of activation in the patient group; it seems likely that it would, but without normal control data we are unable to explore this question. We acknowledge that the lack of control data in this study prevents a full exploration of the interaction between the presence of unilateral pathology and the distribution of activation within and between hemispheres in the patients, in the (limited) sense that we cannot describe how the pattern of activity in patients differs from normal controls. However, addressing this question was not the primary motivation for this study.

Reliability measures based on asymmetry index of mean mTL effect size produced somewhat different findings to those suggested by our analysis of the voxel level mTL activations. Broadly, the Scenes protocols, especially the event‐related analysis, produced the most reliable asymmetry index; Pictures (event) was the least reliable in this context, and the other protocols were between these extremes and moderately reliable. Although a scene encoding task produced good reliability of asymmetry index within the mTL in a prior study [Harrington et al., 2006], in contrast we also found reasonable reliability of asymmetry index for our word encoding task.

Further exploration of the data with ROC analysis found that despite producing reliable asymmetry index results, the Scenes protocols were least able to classify participants by the laterality of seizure onset. The Words (event) protocol was most successful in classifying patients across all sessions, despite relatively weaker reliability of asymmetry index for this protocol compared to the Scenes protocols.

We found that averaging asymmetry data over more than one session led to a modest improvement in patient classification. This was most marked for the Hometown protocol, where classification success was somewhat variable between sessions, reflected in the variability of classification success of this protocol in previous studies, from 64% [Avila et al., 2006] to 90% [Jokeit et al., 2001]. We recognise that the issue of how to measure asymmetry or lateralisation index is complex, with several solutions having been proposed, for example see [Seghier, 2008] and [Wilke and Lidzba, 2007] for further discussion. There remains a need to establish a consensus for conducting asymmetry or lateralisation index measurement to facilitate comparison between studies.

### Strengths of Study

In the past, it has been acknowledged that accurate imaging of mTL structures is technically challenging [Detre, 2004]. However, in the present study we attempted to reduce the effect of susceptibility artefact and signal loss, and in turn maximise our sensitivity to mTL, by selecting thin slices and choosing a field of view resulting in a small in plane voxel size, while still maximising coverage of the hippocampus and neighbouring temporal lobe structures.

Our study has the strengths of addressing reliability in the relevant patient group rather than in normals; and we examined seven protocols across three sessions, making our study one of the larger to be performed. Reliability and reproducibility of fMRI tasks has been the subject of considerable prior investigation. Most studies have involved relatively small groups of normal subjects (*n* < 20) [Aron et al., 2006; Bennett and Miller, 2013; Caceres et al., 2009; Clement and Belleville, 2009; Friedman et al., 2008; Harrington et al., 2006; Loubinoux et al., 2001; Raemaekers et al., 2007; Wagner et al., 2005; Wei et al., 2004] undertaking one or two protocols on two occasions; though a few studies have involved larger groups of normal subjects [Plichta et al., 2012; Putcha et al., 2011; Whalley et al., 2009], three or more fMRI protocols [Clement and Belleville, 2009; Harrington et al., 2006; Plichta et al., 2012] and up to 8 repeated scanning sessions [Wei et al., 2004]. Prior studies in normal subjects show that reliability is very variable between brain regions [Caceres et al., 2009; Friedman et al., 2008; Wei et al., 2004; Whalley et al., 2009] typically being higher in the activated network [Aron et al., 2006]. The nature of the in‐scanner task and the analysis model have an important effect on reliability [Bennett and Miller, 2013; Caceres et al., 2009; Clement and Belleville, 2009]. Whereas reliability for some simpler sensorimotor tasks may be very high in neocortical regions [Friedman et al., 2008; Loubinoux et al., 2001; Raemaekers et al., 2007], memory fMRI studies typically showed lower reliability in mTL, with ICCs typically below 0.5, and sometimes much lower [Clement and Belleville, 2009; Putcha et al., 2011; Wagner et al., 2005]. Furthermore, a verbal memory task may be less reliable than other memory fMRI tasks [Harrington et al., 2006].

Studies of fMRI reliability have been performed less often in clinical groups (patient groups or at‐risk groups). A study of reliability of a language protocol in epilepsy patients showed widespread high reliability within the activated language network [Fernandez et al., 2003]; language fMRI was similarly highly reliable in a group of subjects at‐risk for schizophrenia [Whalley et al., 2009]. To our knowledge, studies of reliability of memory fMRI protocols in clinical groups have only been carried our in mild cognitive impairment [Clement and Belleville, 2009; Zanto et al., 2014] and early Alzheimer's disease [Atri et al., 2011]; these studies showed a considerable range of reliability in an mTL ROI, depending on the in‐scanner task and analysis method, with some choices of methods resulting in high reliability [Atri et al., 2011] and others very low [Clement and Belleville, 2009].

### Limitations of Study

In our study the reliability of our task related signals may have been affected by a number of variables. One factor that may have influenced reliability is variability in behavioural performance across tasks and across participants. We chose not to drop participants from our study on the basis of performance, as it is important not to exclude important sources of variability which may be present in the clinical population and in a clinical setting. In addition, we observed significant differences in behavioural performances for the memory tasks we explored; whilst this may limit the direct comparability of tasks, the relative task reliability we found still has relevant clinical implications. Furthermore, all tasks produced behavioural performances that were significantly different from chance, suggesting that each of the protocols did successfully evoke a meaningful memory effect.

Another potential source of variability which may have impacted on reliability comparison across tasks was the variation in control conditions used for each of the four tasks, as has been argued previously [Harrington et al., 2006]. In our study here, we do not seek to match the tasks for baseline, but simply seek to compare performance of the tasks as previously published. Our findings do not preclude future protocol optimisation, and indeed our findings should provide a useful benchmark against which to compare future optimisations. In addition to very different control conditions between protocols, in our study we also chose not to match the number of alternative forms of stimuli used for each of the sessions. For example, whilst we presented an alternative set of stimuli for each of the three sessions for the Scenes, Words and Pictures protocols, we did not vary the route imagined for the Hometown task. This decision was made as we felt it would have been impossible to match the complexity and difficulty of three imagined walks for each of our participants.

Further, although we did use the robust weighted least squares method [Diedrichsen and Shadmehr, 2005] to detect and adjust for noise and movement artefacts, we chose not to exclude participants from the analysis due to excessive movement, as we wished to ensure the study results were reproducible in future samples of clinical participants. This approach has similarly been adopted by others [Putcha et al., 2011].

## CONCLUSIONS

Any recommendation is context‐specific and based on available evidence. If the question to be addressed is the reliable localization of mTL activity for memory‐related tasks in individual mTLE patients, it could be argued that none of these seven protocols is more than modestly reliable. However, if the question is the more plausible and useful clinical question of the lateralization of mTL activity for memory‐related tasks in individual mTLE patients, the Words (event) protocol described here and used by others is reliable, and the most successful of all seven protocols in classifying mTLE patients by side of seizure onset across all fMRI sessions. We assume these characteristics of the Word (event) protocol are related to its tendency to be strongly activating and strongly lateralising, compared to the other six protocols examined. There was a weak trend that combining data over repeated fMRI sessions improved the classification of patients, hence it might be reasonable to perform the study on more than one occasion.

We anticipate that the data presented here will inform the design and powering of a trial of fMRI in the preoperative prediction of postoperative memory decline in mTLE. We hope that our approach here might contribute to the design of similar comparative studies of other potential clinical applications of fMRI.

## Supporting information

Supplementary Information Figures

Supplementary Information Tables
