# Supplementary material for: Bringing memory fMRI to the clinic: Comparison of seven memory fMRI protocols in temporal lobe epilepsy
Source: Hum Brain Mapp. 2015 Mar 2;36(4):1595–608. doi: 10.1002/hbm.22726 (PMC4855630; doi:10.1002/hbm.22726)
Supplement: Supplementary file 1 — Supplementary Information Figures [file HBM-36-1595-s001.docx]

**Supplementary Figure 1.** Whole brain EPI image for subject EPIMEM01, at first visit (T1). The final sagittal plane indicates the position of coronal slice.

**Supplementary Figure 2.** Anatomical bilateral medial temporal lobe ROI overlaid on a single high resolution EPI image. The final sagittal plane indicates the position of each coronal slices.

**Supplementary Figure 3.** Group t maps (voxelwise threshold p<0.001) for T1 for A) Hometown Walking Task, B) Scenes Task – block design, C) Scenes Task – event design, D) Pictures Task – block design, E) Pictures Task – event Design, F) Words Task –block design and G) Words Task – event design. All activations overlaid on a single subject high resolution EPI image. For each image, the range of t values is set as 0.01-10 to aid comparison across task. The final sagittal plane panel indicates the position of each coronal slice.

**Supplementary Figure 4**: ICC maps for T2 vs. T3 for A) Hometown Walking Task, B) Scenes Task – block design, C) Scenes Task – event design, D) Pictures Task – block design, E) Pictures Task – event Design, F) Words Task –block design and G) Words Task – event design. All ICC maps overlaid on a single subject high resolution EPI image. Thresholded regions above ICC > .60 are shown in red. The final sagittal plane panel indicates the position of each coronal slice.


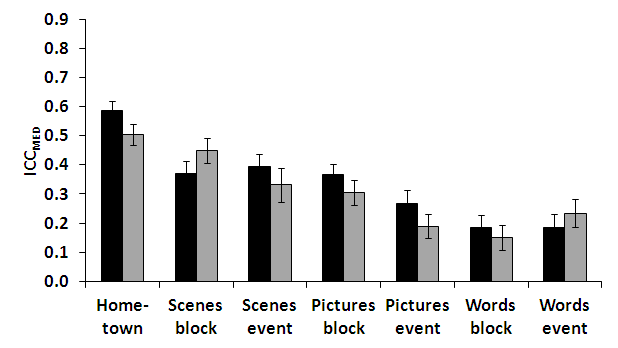


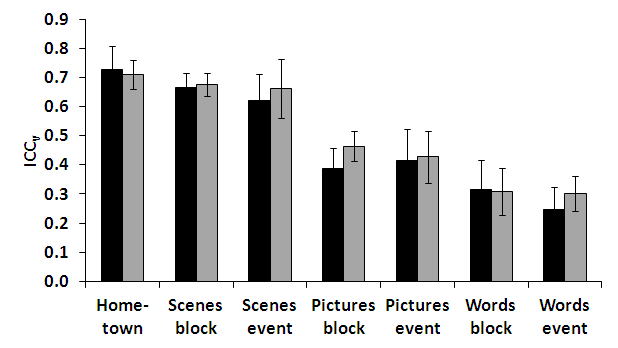


**Supplementary Figure 5.** Reliability of medial temporal lobe ROI BOLD signal activation for each fMRI protocol. Black bars show comparison of T1 with T2, and grey bars show comparison of T2 with T3 (n=16). Error bars show SEM. The upper panel shows ICC_med_ and the lower panel shows ICC_v_.

ICC = intraclass correlation coefficient, ICC_med_ and ICC_v_ are defined in the Methods section.


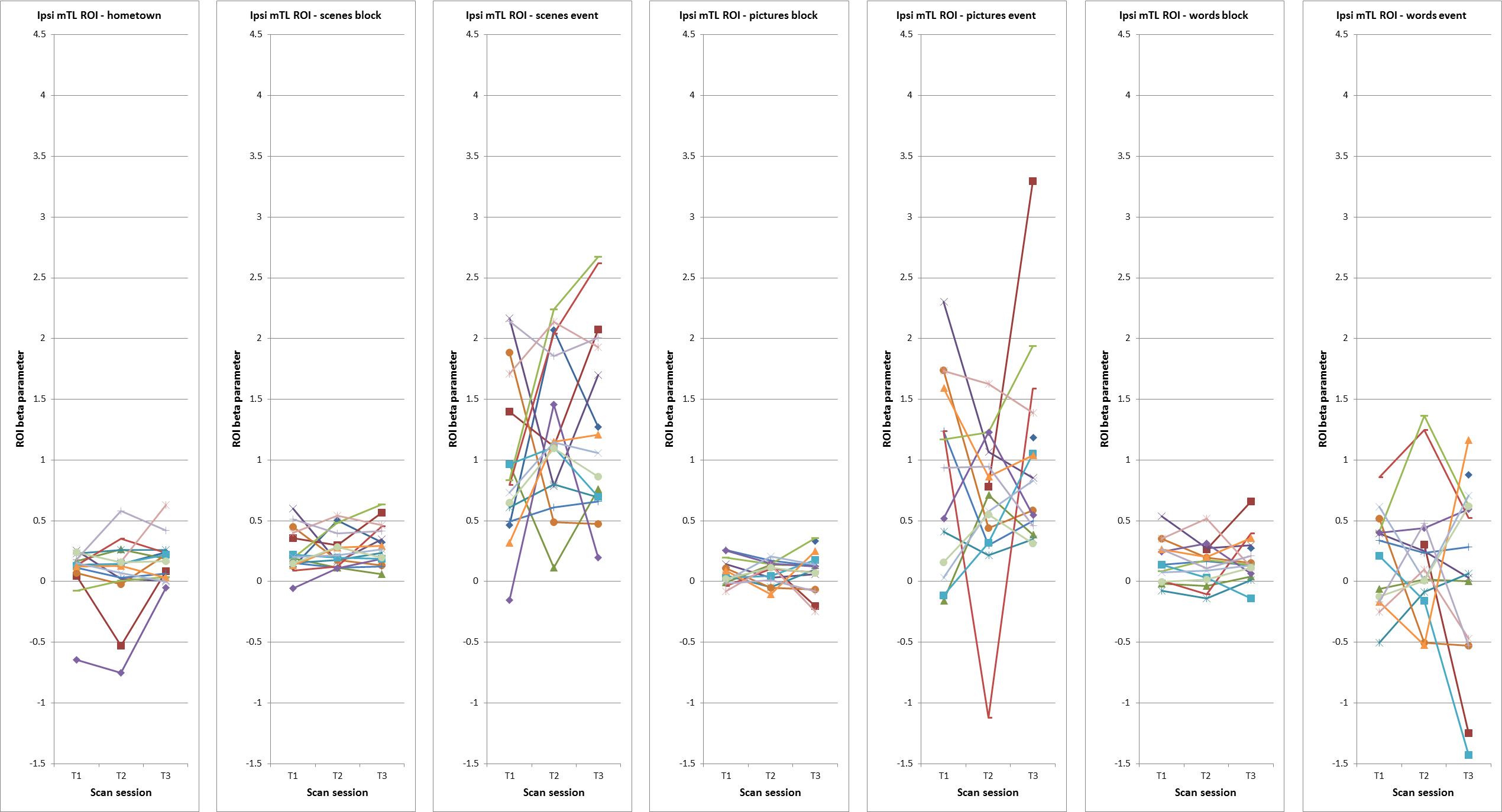


**Supplementary Figure 6.** Mean beta parameter for all voxels in the ipsilateral mTL ROI reaching p < 0,001 uncorrected, for all 16 subjects, and for each protocol and session. Coloured points joined by a line indicate values for a single subject (the same colour used for each subject in each panel here and in Supplementary Figure 7). The panels show data for each protocol – left to right: Hometown, Scenes Block, Scenes Event, Pictures Block, Pictures Event, Words Block, Words event. Note that protocols which show little change in mean beta parameter between sessions tend to have smallest effect sizes. Larger effects sizes are associated with event-related designs, but these seem to be much more variable between sessions.


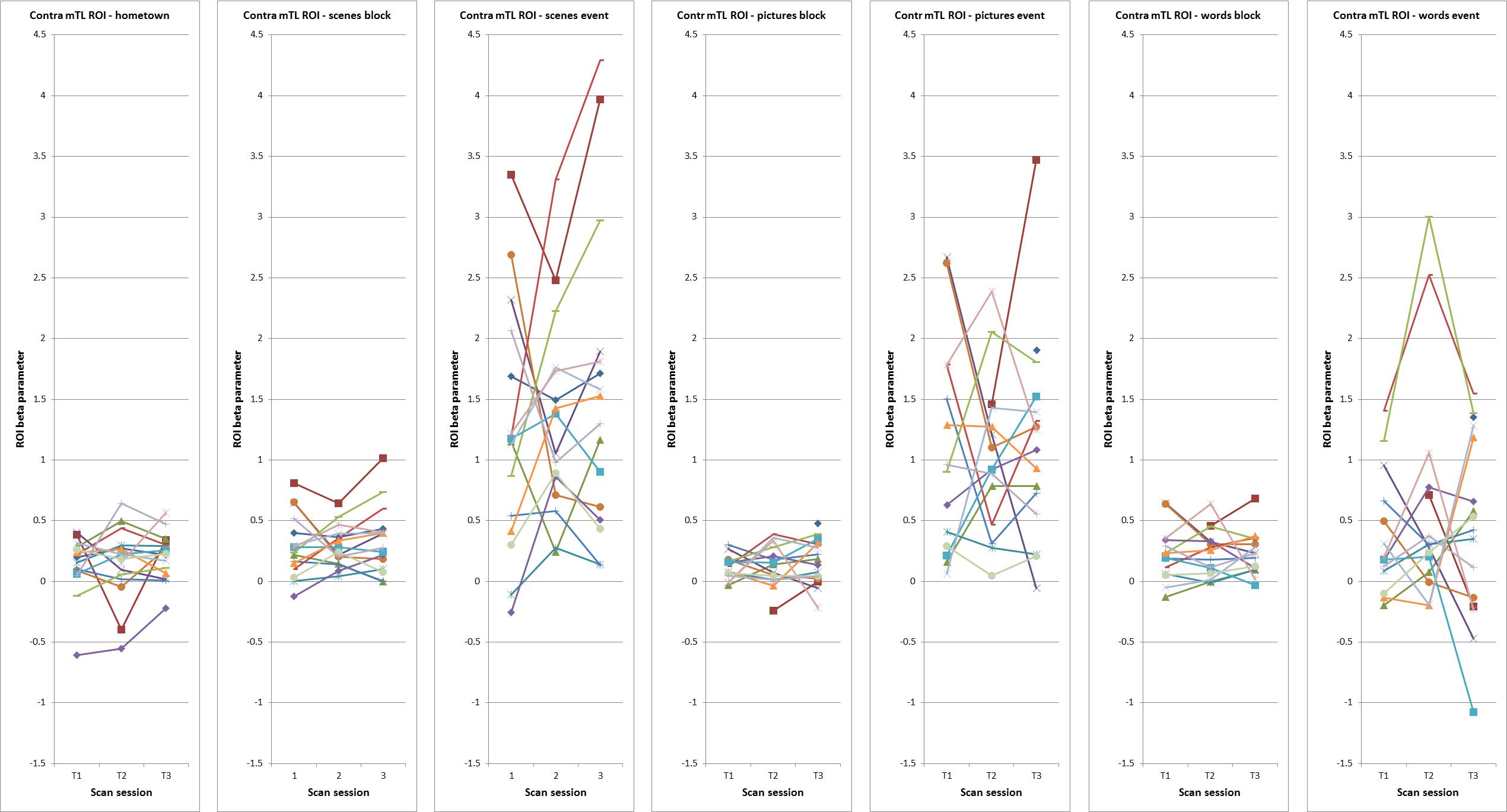


**Supplementary Figure 7.** Mean beta parameter for all voxels in the contralateral mTL ROI reaching p < 0,001 uncorrected, for all 16 subjects, and for each protocol and session. Coloured points joined by a line indicate values for a single subject (the same colour used for each subject in each panel here and in Supplementary Figure 6). The panels show data for each protocol – left to right: Hometown, Scenes Block, Scenes Event, Pictures Block, Pictures Event, Words Block, Words event. Note the similarity with Supplementary Figure 6, but also note a trend to higher effect sizes in the contralateral ROI compared with the ipsilateral. The y-axis scale is the same as Supplementary Figure 6 to aid comparison.


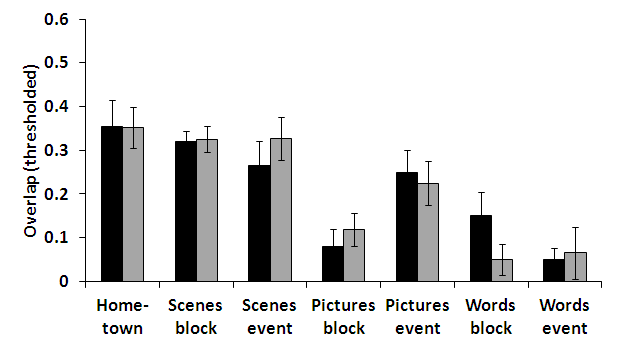


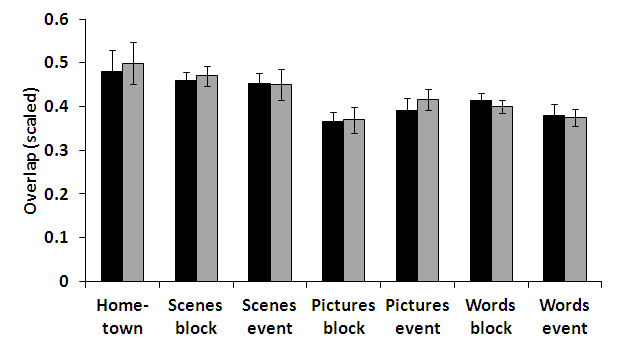


**Supplementary figure 8.** Overlap values of activated voxels in medial temporal lobe ROI for each fMRI protocol (using all available data from n=16). Black bars show overlap values comparing T1 with T2; grey bars show overlap values comparing T2 with T3. The upper panel shows overlap of binary maps of voxels thresholded for significance at p < 0.001, within the MTL ROI, by task; the lower panel shows overlap values generated from raw t-images individually normalised to peak t values within the medial temporal lobe ROI.

**Supplementary Figure 9.** Average laterality index (using all available data from n=16) of mean voxel activity in each medial temporal ROI (average of voxels thresholded at P<0.001) for each fMRI protocol and session. Error bars show SEM. Upper left panel shows laterality index as right-minus-left ROI for all patients, which may provide an indication of material-specific lateralisation of the fMRI tasks; lower left panel shows laterality index as contralateral-minus-ipsilateral ROI for all patients; upper right panel shows laterality index as contralateral-minus-ipsilateral ROI for left-onset patients (note this is the same as right-minus-left ROI); lower right panel shows laterality index as contralateral-minus-ipsilateral ROI for right-onset patients (note this is the same as left-minus-right ROI);. Black bars are for T1, white bars T2 and grey bars T3.


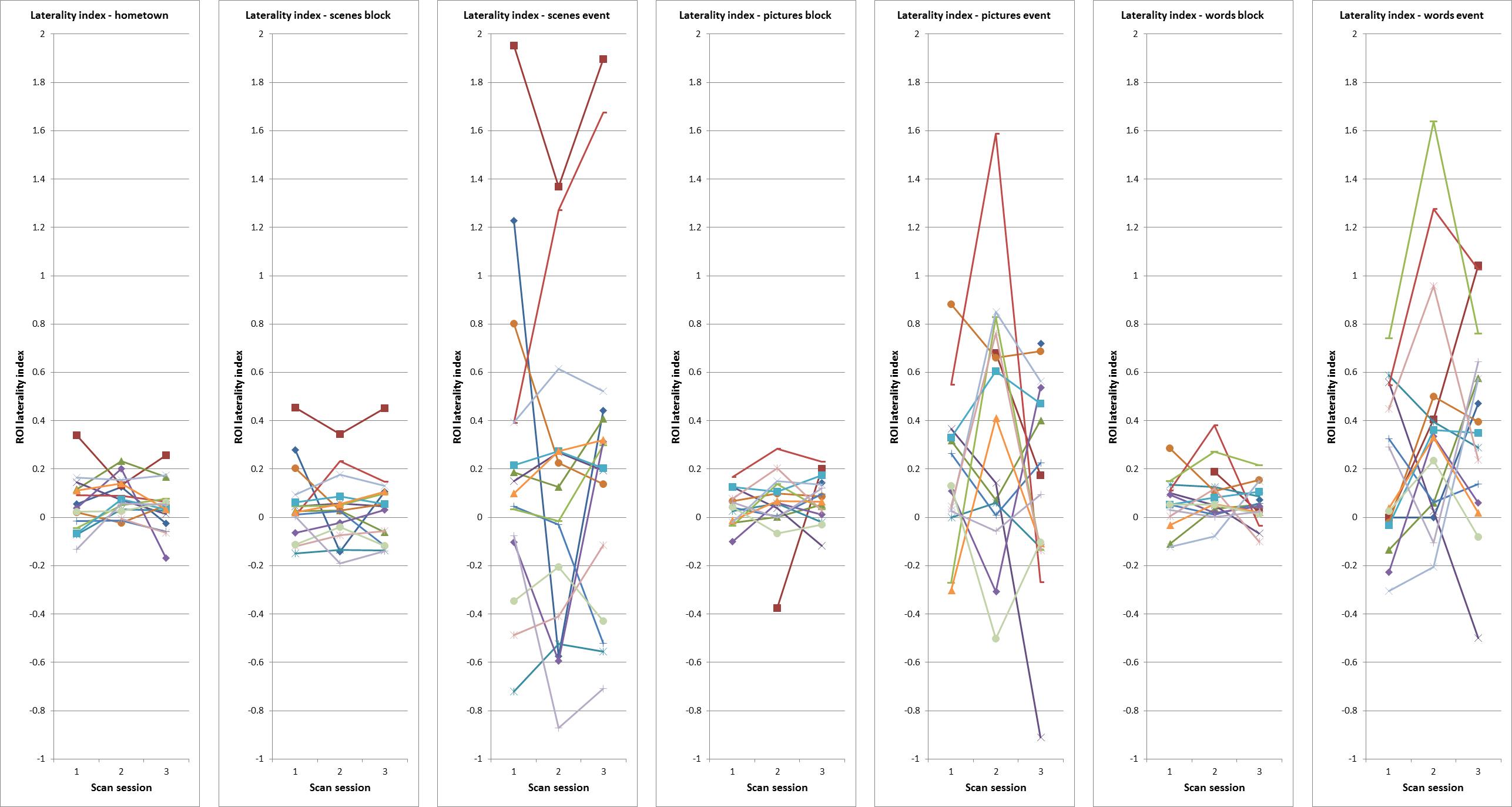


**Supplementary Figure 10.** Laterality index for mean beta parameter for all voxels in the mTL ROI reaching p < 0,001 uncorrected, for all 16 subjects, and for each protocol and session. Coloured points joined by a line indicate values for a single subject (the same colour used for each subject in each panel here and in Supplementary Figures 6 and 7). The panels show data for each protocol – left to right: Hometown, Scenes Block, Scenes Event, Pictures Block, Pictures Event, Words Block, Words event. . Asymmetry index >0 indicates that activity is greater in the contralateral ROl; asymmetry index <0 indicates that activity is greater in the ipsilateral ROI.

**Supplementary figure 11.**: Marginal distribution for T2 vs T3 (n=13) for the whole brain and the task related activated network (as defined in T1 data only), for thresholds of 3.93 for A) Hometown, B) Scenes Block, C) Scenes Event, D) Pictures Block, E) Pictures Event, F) Words Block and G) Words Event.
