# Supplementary material for: Bringing memory fMRI to the clinic: Comparison of seven memory fMRI protocols in temporal lobe epilepsy
Source: Hum Brain Mapp. 2015 Mar 2;36(4):1595–608. doi: 10.1002/hbm.22726 (PMC4855630; doi:10.1002/hbm.22726)
Supplement: Supplementary file 2 — Supplementary Information Tables [file HBM-36-1595-s002.docx]

| **Subject** | **Age** | **Gender** | **NART FSIQ** | **Type of Seizures** | **Frequency of Seizures** | **Age of Onset (years)** | **Laterality** | **MRI** | **Medications** |
| --- | --- | --- | --- | --- | --- | --- | --- | --- | --- |
| 1 | 45 | M | 115 | CPS | 1-2 per week | 15 | Right | Right mesial temporal sclerosis | Tegretol, Levetiracetam |
| 2 | 19 | F | 100 | CPS | 1 per week | 3 | Right | Right mesial temporal sclerosis | Topiramate, Lamotrigine, Levetiracetam, Clobazam |
| 3 | 49 | F | 120 | CPS and GTCS | CPS 1 per month, GTCS 1 per month | 7 | Right | Right mesial temporal sclerosis | Topiramate, Carbamazepine |
| 4 | 38 | F | 120 | CPS, GTCS | 4 per year | Not known | Right | Right mesial temporal sclerosis | Levetiracetam, Lamotrigine, Clobazam |
| 5 | 43 | M | 121 | CPS | 1 per day | 25 | Left | Non lesional left temporal lobe epilepsy. FDG PET reduced uptake in left temporal lobe. | Tegretol , Levetiracetam, Topiramate |
| 6 | 57 | F | 114 | SPS, CPS | SPS up to 25 per day, CPS 1 every few months | 28 | Left | Non lesional temporal lobe epilepsy. Ictal EEG shows left anterior temporal onset. | Carbamazepine |
| 7 | 39 | F | 101 | CPS | 3-13 per month | 20 | Left | Left mesial temporal sclerosis | Lamotrigine, Tegretol |
| 8 | 59 | F | 105 | CPS, GTCS | 4-5 per month | 10 | Left | Left mesial temporal sclerosis | Carbamazepine, Phenytoin, Zonisamide, Clobazam |
| 9 | 59 | M | 118 | CPS, GTCS | 1-2 per year | 45 | Left | Left mesial temporal sclerosis | Clobazain, Sodium Valporate |
| 10 | 51 | M | 111 | CPS, GTCS | 9-10 per month | 8 | Right | Right mesial temporal sclerosis and right MTL benign tumour. | Lamotrizine, Phenytoin, Levetiracetam |
| 11 | 44 | M | 114 | CPS | 1 per week | 15 | Right | Right mesial temporal sclerosis | Tegretol, Lamotrigine, Citalopram, Olazapine |
| 12 | 33 | F | 108 | CPS, GTCS | 7 per year | 12 | Right | Right mesial temporal sclerosis | Lamotrigine, Venlafaxine, Pregabalin |
| 13 | 29 | F | 115 | CPS | 1 per week | 15 | Right | Post traumatic lesion, right amygdale | Tegretol, Lamotrigine, Citalopram, Olanzapine |
| 14 | 49 | M | 123 | CPS | 4-5 per week | 2 | Left | Left mesial temporal sclerosis | Phenytoin, Levetiracetam, Toprimate, Carbamazepine, Sodium Valporate |
| 15 | 55 | F | 123 | SPS | Seizure free for >12 months | 40 | Right | Bilateral mesial temporal sclerosis. Right more affected than left | Tegretol, lansoprazole |
| 16 | 54 | F | 112 | SPS, CPS | Up to 12 per month | 44 | Bilateral | High signal right hippocampus. Ictal EEG shows bilateral temporal onset. | Topiramate, lacosamide |

**Supplementary** **Table I:** Demographic and clinical data for the 16 participants.

CPS = complex partial seizure; GTCS = generalised tonic-clonic seizure; SPS = simple partial seizure; FDG PET = 18F-deoxyglucose positron emission tomography.

| **Subject** | **Scenes** | | | | | | **Words** | | | | | | **Pictures** | | | | | |
| --- | --- | --- | --- | --- | --- | --- | --- | --- | --- | --- | --- | --- | --- | --- | --- | --- | --- | --- |
|  | **T1** | | **T2** | | **T3** | | **T1** | | **T2** | | **T3** | | **T1** | | **T2** | | **T3** | |
|  | **Miss** | **Like** | **Miss** | **Like** | **Miss** | **Like** | **Miss** | **Like** | **Miss** | **Like** | **Miss** | **Like** | **Miss** | **Like** | **Miss** | **Like** | **Miss** | **Like** |
| 3 | 5 | 70 | 5 | 43 | 0 | 43 | 3 | 66 | 0 | 61 | 1 | 58 | 1 | 68 | 3 | 59 | 1 | 55 |
| 4 | 2 | 68 | 2 | 61 | 0 | 71 | 0 | 47 | 6 | 70 | 27 | 73 | 3 | 59 | 1 | 67 | 16 | 64 |
| 5 | 0 | 76 | 0 | 48 | 0 | 62 | 0 | 51 | 0 | 56 | 1 | 67 | 3 | 62 | 0 | 63 | 0 | 66 |
| 6 | 5 | 52 | 17 | 44 | 13 | 44 | 7 | 55 | 6 | 44 | 26 | 65 | 14 | 57 | 6 | 39 | 27 | 67 |
| 7 | 0 | 49 | 0 | 63 | 0 | 78 | 0 | 60 | 0 | 59 | 0 | 69 | 0 | 61 | 0 | 69 | 0 | 76 |
| 8 | 32 | 86 | 44 | 69 | 51 | 94 | 46 | 76 | 9 | 63 | 16 | 68 | 26 | 85 | 34 | 74 | 21 | 71 |
| 9 | 2 | 63 | 2 | 68 | 2 | 65 | 4 | 52 | 1 | 55 | 0 | 61 | 1 | 75 | 0 | 77 | 0 | 77 |
| 10 | 0 | 30 | 3 | 43 | 0 | 51 | 1 | 57 | 0 | 43 | 0 | 43 | 0 | 44 | 0 | 24 | 0 | 17 |
| 11 | 0 | 94 | 11 | 98 | 3 | 90 | 1 | 65 | 16 | 81 | 1 | 67 | 0 | 73 | 20 | 75 | 0 | 69 |
| 12 | 2 | 61 | 0 | 59 | 2 | 48 | 1 | 49 | 0 | 63 | 0 | 57 | 1 | 59 | 1 | 59 | 1 | 45 |
| 13 | 0 | 65 | 0 | 71 | 0 | 67 | 0 | 66 | 0 | 67 | 0 | 69 | 0 | 57 | 0 | 63 | 0 | 74 |
| 15 | 5 | 68 | 0 | 27 | 0 | 59 | 0 | 50 | 0 | 70 | 0 | 57 | 0 | 56 | 0 | 44 | 0 | 46 |
| 16 | 8 | 60 | 5 | 63 | 2 | 40 | 0 | 43 | 0 | 39 | 1 | 46 | 0 | 40 | 0 | 39 | 0 | 21 |

**Supplementary** **Table II:** In-scanner behavioural data.

Miss = number of stimuli with no button press response. Like = number of stimuli with “like” response.

|  | **T1 compared with T2** | | **T1 compared with T2** | |
| --- | --- | --- | --- | --- |
| **fMRI protocol** | **ICC_(3,1)_** | **Significance** | **ICC_(3,1)_** | **Significance** |
| Hometown | 0.515 | 0.021 | 0.361 | 0.085 |
| Scenes-Block | 0.546 | 0.014 | 0.786 | 0.000 |
| Scenes-Event | 0.573 | 0.010 | 0.795 | 0.000 |
| Pictures-Block | 0.534 | 0.017 | 0.299 | 0.130 |
| Pictures-Event | 0.356 | 0.106 | 0.126 | 0.327 |
| Words-Block | 0.534 | 0.017 | 0.299 | 0.130 |
| Words-Event | 0.562 | 0.018 | 0.710 | 0.002 |

**Supplementary** **Table III.** Reliability of medial temporal lobe ROI BOLD signal laterality index for each fMRI protocol.

T1 is compared with T2, and T2 with T3 (n=15). ICC = intraclass correlation coefficient, ICC_(3,1)_ is defined in the Methods section. Significance threshold set according to false discovery rate.

|  | **T1** | | **T2** | | **T3** | | **Average T1 & T2** | | **Average T1, T2 & T3** | |
| --- | --- | --- | --- | --- | --- | --- | --- | --- | --- | --- |
| **fMRI protocol** | **AUC** | **Sig.** | **AUC** | **Sig.** | **AUC** | **Sig.** | **AUC** | **Sig.** | **AUC** | **Sig.** |
| Hometown | 0.800 | 0.072 | 1.000 | 0.003 | 0.690 | 0.253 | 0.956 | 0.006 | 0.956 | 0.006 |
| Scenes – Block | 0.524 | 0.886 | 0.690 | 0.253 | 0.500 | 1.000 | 0.667 | 0.289 | 0.648 | 0.346 |
| Scenes – Event | 0.571 | 0.668 | 0.595 | 0.568 | 0.619 | 0.475 | 0.595 | 0.568 | 0.595 | 0.568 |
| Pictures – Block | 0.810 | 0.063 | 0.952 | 0.007 | 0.852 | 0.025 | 1.000 | 0.003 | 1.000 | 0.003 |
| Pictures – Event | 0.833 | 0.046 | 0.905 | 0.015 | 0.722 | 0.157 | 0.929 | 0.010 | 0.905 | 0.015 |
| Words – Block | 0.857 | 0.032 | 0.976 | 0.004 | 0.778 | 0.077 | 0.929 | 0.010 | 0.952 | 0.007 |
| Words - Event | 0.905 | 0.015 | 0.976 | 0.004 | 0.926 | 0.007 | 0.952 | 0.007 | 1.000 | 0.003 |

**Supplementary** **Table IV.** Classification of patients into right-onset and left-onset using Receiver Operating Characteristic.

ROC was applied to the laterality index for each subject, for each fMRI protocol and session (n=15). To examine whether repeating a protocol on more than one session contributes to accuracy of lateralisation, asymmetry values were averaged across T1 and T2 for each subject and protocol, and averaged across T1, T2 and T3 for each subject and protocol. AUC = area under curve.
